# Deep Brain Stimulation Increases Seizure Threshold by Altering REM Sleep and Delta Powers During NREM Sleep

**DOI:** 10.3389/fneur.2020.00752

**Published:** 2020-08-12

**Authors:** Hsin-Tzu Tseng, Yi-Tse Hsiao, Pei-Lu Yi, Fang-Chia Chang

**Affiliations:** ^1^Department of Veterinary Medicine, School of Veterinary Medicine, National Taiwan University, Taipei, Taiwan; ^2^Department of Sport Management, College of Tourism, Leisure and Sports, Aletheia University, Taipei, Taiwan; ^3^Graduate Institute of Brain and Mind Sciences, College of Medicine, National Taiwan University, Taipei, Taiwan; ^4^Graduate Institute of Acupuncture Science, College of Chinese Medicine, China Medical University, Taichung City, Taiwan; ^5^Department of Medicine, College of Medicine, China Medical University, Taichung City, Taiwan

**Keywords:** anterior nucleus of thalamus, deep brain stimulation, delta powers during NREM sleep, epilepsy, REM sleep, PTZ kindling

## Abstract

We previously demonstrated that seizure occurrences at different zeitgeber times alter sleep and circadian rhythm differently. On the other hand, the synchronized delta wave of electroencephalogram (EEG) during non-rapid eye movement (NREM) sleep facilitates seizure, while the desynchronized EEG of rapid eye movement (REM) sleep suppresses it. We also elucidated that unilateral deep brain stimulation (DBS) of the anterior nucleus of thalamus (ANT) suppresses seizure recurrence. In the present study, we intraperitoneally injected pentylenetetrazol (PTZ, 40 mg/kg) for 14 consecutive days (PTZ kindling) to induce spontaneous seizure in rats, and a 30-min (delivered 10 min before each PTZ injection) or a 3-h DBS of unilateral ANT (delivered 1 h before each PTZ injection) was applied to suppress seizure. The frequency of DBS stimulation was 200 Hz and the electrical current consisted of biphasic square pulses with 50-μA intensity, 100-μs pulse width, and 4.1-ms stimulation interval. Our results found that PTZ-induced spontaneous seizure did not cause a significant change in the quantity of NREM sleep but suppressed the amount of REM sleep. Unilateral ANT DBS prolonged the onset latency of ictal seizure, decreased the spontaneous seizure duration, and increased the survival rate but did not change the amplitude of epileptiform EEGs during ictal period. Unilateral ANT DBS did not significantly alter NREM sleep but increased the amount of REM sleep. An analysis of the spectrograms of fast Fourier transform indicated that the intensities of all frequencies were enhanced during the PTZ-induced ictal period and the subsequent spontaneous seizure. Thirty minutes of unilateral ANT DBS suppressed the augmentation of low-frequency (<10 Hz) intensities during the spontaneous seizure induced by PTZ kindling. We further found that consecutive injections of PTZ progressively increased the enhancement of the delta powers during NREM sleep, whereas unilateral ANT DBS inhibited this progressive enhancement. It was also noticed that 30 min of ANT DBS exhibited a better efficacy in epilepsy suppression than 3 h of ANT DBS. These results elucidated that unilateral ANT DBS enhanced the seizure threshold by increasing the amount of REM sleep and decreasing the progressive enhancement of delta power during NREM sleep to suppress spontaneous seizure recurrences in PTZ kindling-induced epileptic rats.

## Introduction

The reciprocal influence between epilepsy and sleep has been demonstrated clinically and in animal experiments. Sleep disturbance, either daytime somnolence or insufficient nocturnal sleep, is common in patients with epilepsy (1–4). The causes of sleep disruptions could be the nature of epileptogenesis, the medication of anti-epileptic drugs (AEDs), or both (1). This diversity phenomenon is also found in animal experiments. Electrical kindling-induced epilepsy increases wakefulness and light sleep (5); however, increase of deep slow wave sleep (SWS) and suppression of wakefulness have also been documented in animal experiments (6). These diverse and conflicting outcomes of sleep disturbances in patients with epilepsy or in epileptic animals may be due to the occurrence of seizure at different zeitgeber times (ZTs). A zeitgeber is any external or environmental cue that entrains the animal biological rhythm to a 24:24-h light/dark cycle. We previously applied a rapid kindling protocol in amygdala to induce seizure occurrence specifically at particular ZTs and found that seizure occurs at ZT0, referring to the beginning of the light period, and decreases rapid eye movement (REM) sleep and non-REM (NREM) sleep during the light period in rats. The changes of sleep caused by ZT0 seizure is mediated by the corticotropin-releasing hormone. ZT13 is the beginning of the dark period; seizure, on the other hand, enhances interleukin-1 (IL-1) and increases NREM sleep during the dark period. Both ZT0 and ZT13 seizures do not alter the circadian rhythm of sleep–wake fluctuation. Surprisingly, seizure occurs at ZT6, the middle of the light period, shifting the expression of PER1 protein in the suprachiasmatic nucleus of the hypothalamus, and alters the sleep–wake circadian rhythm, which is mediated by hypocretin (7). This result elucidates that the occurrence of seizure at different ZTs alters sleep homeostasis and circadian rhythm differently in rats.

The synchronous electroencephalogram (EEG) oscillations during SWS, combined with the sleep spindles and K complexes during the N2 stage of NREM sleep, promote the propagation of epileptiform EEGs. Asynchronous EEG oscillations during wakefulness and REM sleep, on the other hand, suppress epileptogenesis (8). There is a vicious cycle created by the epilepsy-induced sleep disruptions, which in turn leads to worsening epilepsy. Therefore, correction of sleep disruptions should be considered as part of the therapy in epilepsy management.

About 70% of patients with epilepsy can be controlled by currently available AEDs. However, there are still 30% of patients who do not respond to AEDs despite a careful administration of the optimized dose (9). Resective surgery is an alternative therapy for refractory epilepsy, but not all patients with refractory epilepsy are suitable for surgery. Vagus nerve stimulation (10) and deep brain stimulation (DBS) (11, 12) have been considered for treating refractory epilepsy. DBS is primarily used to treat movement disorders, such as tremor and Parkinson's disease (PD) (13). DBS exhibits a certain efficacy in seizure suppression according to the literatures and our previous findings (12, 14–16), and DBS has further been approved by the US Food and Drug Administration as an add-on treatment for focal epilepsy (17). A feasible target for DBS is always crucial. For example, DBS of the subthalamic nucleus has been used for improving the essential features of PD, and stimulation of pedunculopontine nucleus may ameliorate the gait disorders in PD (18). The anterior nucleus of thalamus (ANT) seems to be a promising target for DBS to suppress epilepsy in animal (14, 19) and human studies (11, 12). Our previous study had demonstrated that the electrical stimulation of unilateral ANT with high-frequency and low-intensity currents reduces the rate of pilocarpine-induced epilepsy in rats (16). Nevertheless, the underlying mechanisms of DBS remain unclear, although several hypotheses have been proposed. The potential mechanisms for DBS to suppress epilepsy include the following: (1) Stimulation of the axon causes antidromic action potentials to collide the intrinsic action potentials (20); (2) DBS induces a depolarization blockade in soma to subsequently inactivate voltage-gated sodium channels, increase extracellular potassium concentrations, and silence neurons (21); and (3) DBS activates astrocytes and releases adenosine to suppress neuronal activity (22, 23). However, as we aforementioned that correction of sleep disruptions should be considered as part of the therapy in epilepsy management, herein we investigated whether unilateral ANT DBS alters the features of sleep to increase seizure threshold and consequently exhibits its therapeutic efficacy on seizure suppression.

## Materials and Methods

### Substances

A stock solution of pentylenetetrazol (PTZ, Sigma-Aldrich, St. Louis, MO, USA) was dissolved in pyrogen-free solution (PFS) and stored at −20°C until administration. The dose of PTZ used in the current experiment to induce spontaneous seizure recurrence was 40 mg/kg by intraperitoneal (i.p.) injection, and the rats received i.p. injection once a day at the beginning of the dark period, which lasted for 14 days (PTZ kindling).

### Animals

Male Sprague–Dawley rats (250–300 g; BioLASCO Taiwan Co., Ltd.) were used in this study. These rats were anesthetized with Zoletil® (50 mg/kg, Carros, France) and xylazine (7.4 mg/kg, Sigma-Aldrich). All rats were surgically implanted with three EEG screw electrodes on the right frontal and parietal lobes and the left occipital lobe and two electromyogram (EMG) electrodes into the neck muscle as previously described (16, 24). A concentric bipolar electrode (O.D. 0.125 mm, FHC, Bowdoinham, ME, USA) used for DBS was implanted directly into the left ANT (coordinates: AP, −2.0 mm from bregma; ML, 1.5 mm; DV, 5.5 mm) according to our previous study (16). The insulated leads from the EEG and the EMG electrodes were plugged into a Teflon pedestal (Plastics One, Roanoke, VA, USA) and fixed with the DBS stimulation electrode together on the skull by dental acrylic (Tempron, GC Co., Tokyo, Japan). The incision was topically treated with neomycin sulfate ointment (Genuine Chemical Pharmaceutical Co., Ltd., Taiwan) and the rats received an i.p. injection of antibiotic (penicillin G benzathine, 6,000 IU, Sigma-Aldrich) to prevent infection. The analgesic ibuprofen (0.4 g/250 ml) was added into the drinking water, and the rats can drink freely for 5 consecutive days after surgery. We allowed the rats to recover from surgery for 1 week before the experimental manipulations. The rats were kept separately in an individual recording cage with the temperature maintained at 23–24°C, and the light/dark cycle in the animal room was controlled in at 12:12 h cycle. All food and water were available *ad libitum*. At 3 days before the baseline recording, the rats were connected to the EEG recording device *via* a flexible cable. All these procedures were approved by the National Taiwan University Institutional Animal Care and Use Committee (approval number NTU106-EL-00102).

### Recording Apparatus

The signals acquired from the EEG and the EMG electrodes were fed into an amplifier (Colbourn Instruments, Lehigh Valley, PA, USA; model V75-01). The EEG and the EMG were, respectively, amplified with a gain of 10,000 and 5, 000, and analog bandpass was filtered between 0.1 and 40 Hz for EEG and between 90 and 1 kHz for EMG. These filtered EEG and EMG signals were transmitted to analog-to-digital conversion (NI PCI-6033E, National Instrument, Austin, TX, USA) with a sampling rate of 128 Hz. The digitized EEGs and EMGs were stored as binary files for subsequent seizure and sleep analyses.

### Unilateral ANT DBS Stimulation

The DBS stimulation electrode was connected to a stimulus generator (STG4002, Multi Channel Systems MCS GmbH, Germany), and stimulation current, with a frequency of 200 Hz, pulse width of 90 μs, interval of 4.1 ms, and intensity of 50 μA, was delivered into the left ANT as we have previously described (16). The ANT stimulation was started either 10 min or 1 h before each time of PTZ injection and lasted for 30 min (30-min ANT DBS) or 3 h (3-h ANT DBS). The ANT stimulation artifact would not be detected because of the low sampling rate of the EEG recording. The ANT stimulation did not alter the quantity of the EEG signals as we have observed in our previous study (16).

### Analysis of Seizure Activity

**Figure 1:**
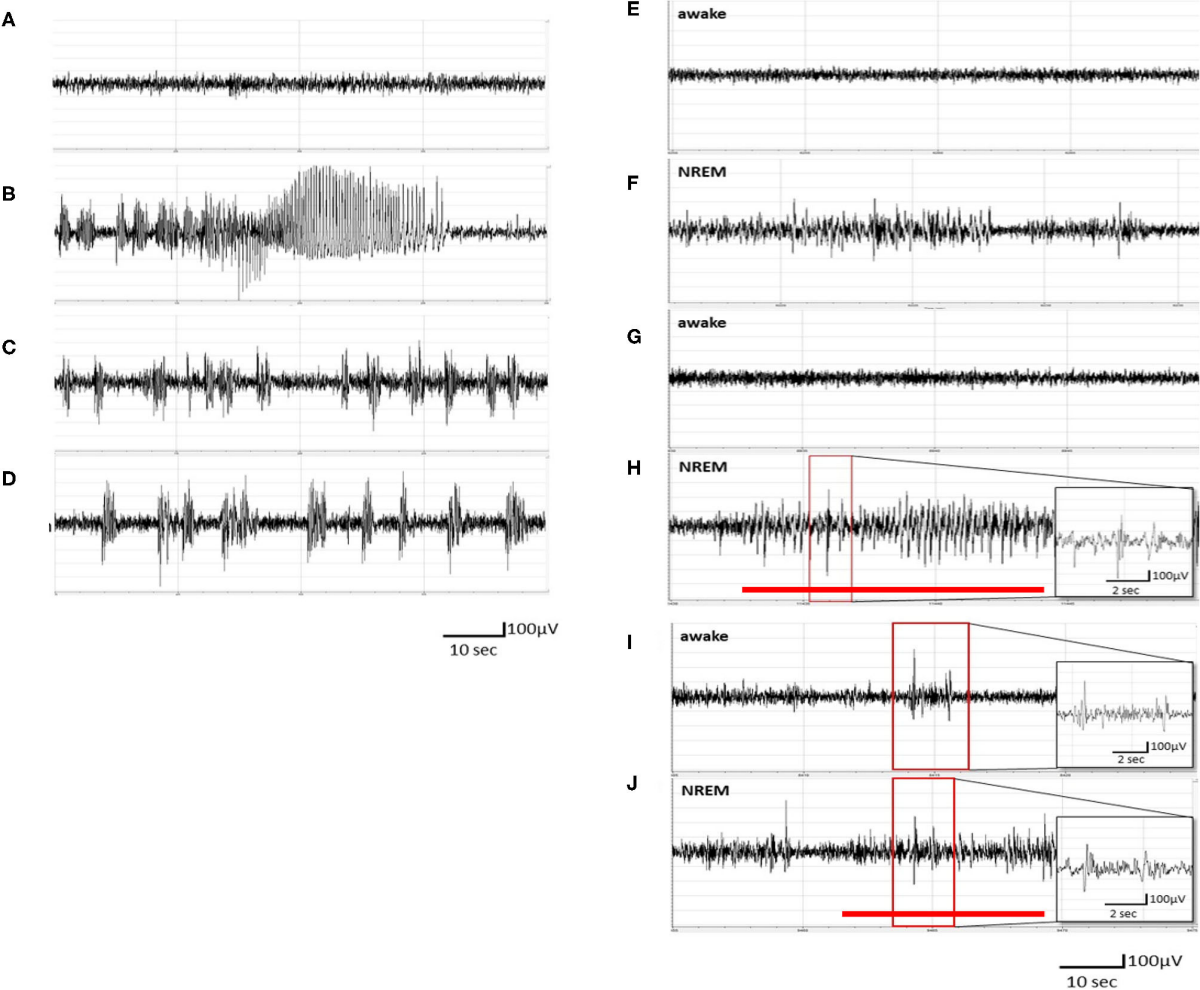
Pentylenetetrazol (PTZ) kindling-induced ictal epileptiform electroencephalogram (EEG) and spontaneous recurrence of epileptiform EEGs. **(A)** The EEGs obtained after the vehicle (pyrogen-free solution) injection. **(B)** The ictal epileptiform EEGs obtained after the 1st PTZ injection. **(C)** The ictal epileptiform EEGs obtained after the 7th PTZ injection. **(D)** The ictal epileptiform EEGs acquired after the 14th PTZ injection. **(E,F)** The EEGs obtained 6 h after the 1st PTZ injection during wakefulness and NREM sleep, respectively. **(G,H)** The EEGs obtained 6 h after the 7th PTZ injection during wakefulness and NREM sleep, respectively. **(I,J)** The EEGs obtained 6 h after the 14th PTZ injection during wakefulness and NREM sleep, respectively. The red line depicts the duration for spontaneous recurrence of epileptiform EEGs.

We determined the epilepsy duration and the onset latency of ictal seizure by visual scoring using a custom software (ICELUS, M. Opp) written in LabView for Windows (National Instrument). The onset latency of ictal seizure was defined as the time period between the PTZ injection and the first observation of ictal epileptiform EEG activity. Since we administered a low dose of PTZ (40 mg/kg) to progressively induce kindled epilepsy, the ictal seizure with a higher amplitude occurred after injection and lasted for about 100 s (e.g., Figures 1B–D, 3B,C; marked with a red line). The consequent epileptiform activities with a relatively low amplitude after the ictal period are defined as the spontaneous seizure activities (e.g., Figures 1I,J, 3B,C) throughout the rest of 24 h. The fast Fourier transform (FFT) and multi-taper time–frequency spectrum of the EEGs were analyzed by the Metlab Signal Processing Toolkit provided by the open-source Chronux algorithms (http://chronux.org/). Every 12-s epoch was used for the power analysis of the EEG spectrum. The power of the EEG spectrum was determined during the following 20 min after the PTZ injection, including the ictal period and before and after the ictal seizure.

### Analysis of Sleep–Wake Activity

The vigilance states of animals were determined by visual scoring of 12-s epochs using a custom software ICELUS. The animal's behavior was classified as either NREM sleep, REM sleep, or waking based on previously defined criteria (24). Briefly, NREM sleep is characterized by large and synchronous EEG slow waves, dominant power density values in the delta frequency band (0.5–4.0 Hz), and progressively reduced muscle tone. During REM sleep, the amplitude of the EEG is reduced, the theta frequency (6.0–9.0 Hz) of EEG becomes dominant, and the muscle tone is the lowest. During wakefulness, the muscle tone is the highest and the amplitude of EEG is similar to that observed during REMS, but the power density values in the delta frequency band are generally greater than those in the theta frequency band.

### Experimental Procedures

A total of 45 Sprague–Dawley rats were used and divided into three groups: the PTZ group (*n* = 22), 30-min ANT DBS + PTZ group (*n* = 15), and 3-h ANT DBS + PTZ group (*n* = 8). The final sample size for each group is less than the original sample size because some animals died after the full PTZ kindling. In order to reduce the number of animals used in the current study, we did not repeatedly perform the sham surgery + PTZ group with a DBS electrode implantation but without stimuli because the DBS electrode implantation exhibits no effect on seizure suppression according to our previous study (16). In the PTZ group, the rats were given an i.p. injection of PFS (the vehicle) as the control and subsequently received an i.p. injection of 40 mg/kg PTZ at the beginning of the dark period once a day and which lasted for 14 days to initiate ictal seizure and induce spontaneous seizure recurrence. In the 30-min ANT DBS + PTZ group, the rats received DBS stimuli 10 min prior to the PTZ injection and which lasted for 30 min. The rats in the 3-h ANT DBS + PTZ group were delivered with DBS stimuli 1 h before the PTZ injection, and the stimuli was continued for 3 h. The rats from these three groups were sacrificed at the end of the experiment, and brain tissues were collected to identify the trace of the DBS electrode location. Only the rats with correct DBS electrode location were used for further analysis.

### Statistical Analyses

All values acquired from the EEG recordings, spectra, and sleep–wake activities were depicted as the mean ± standard error of mean (SEM) for the indicated sample sizes. The powers of the spectra were represented as the mean with 95% confidence interval. The difference between groups was analyzed with one-way analysis of variance (ANOVA) with a *post-hoc* comparison of Scheffé test. The survival rate was analyzed with the chi-square test. An α level <0.05 was taken to indicate a statistically significant difference.

## Results

### PTZ Kindling Induces Spontaneous Recurrence of Epileptiform EEGs

No epileptiform EEG was observed in unmanipulated rats treated with an i.p. injection of vehicle (Figure 1A). The rats exhibited ictal epileptiform EEGs and behaviorally demonstrated a tonic–clonic seizure after receiving the 1st PTZ injection (40 mg/kg, i.p.; Figure 1B). The ictal epileptiform EEGs and seizure behaviors occurred during the first 6 h after the PTZ injection, and no spontaneous epileptiform EEGs could be detected in the brain waves of NREM sleep and wakefulness at 6 h after the 1st administration of PTZ (Figures 1E,F). The ictal period is defined as the epileptiform EEG with an amplitude higher than 2 V and which lasted for about 100 s. The severity of ictal epileptiform EEGs and seizure behaviors were reduced in the subsequent PTZ injections; for example, Figure 1C demonstrated the epileptiform EEGs acquired immediately after the 7th PTZ injection and Figure 1D elicited the EEGs after the 14th PTZ injection. Spontaneous spike-and-wave epileptiform EEGs could be detected during NREM sleep at 6 h after the 7th PTZ injection (Figure 1H), although no spontaneous epileptiform EEG was found during wakefulness (Figure 1G). Nevertheless, spontaneous epileptiform EEGs were found in both wakefulness and NREM sleep after the rats received the 14th PTZ injection (Figures 1I,J).

### Effects of Unilateral ANT DBS on the Severity of PTZ-Induced Seizure

**Figure 2:**
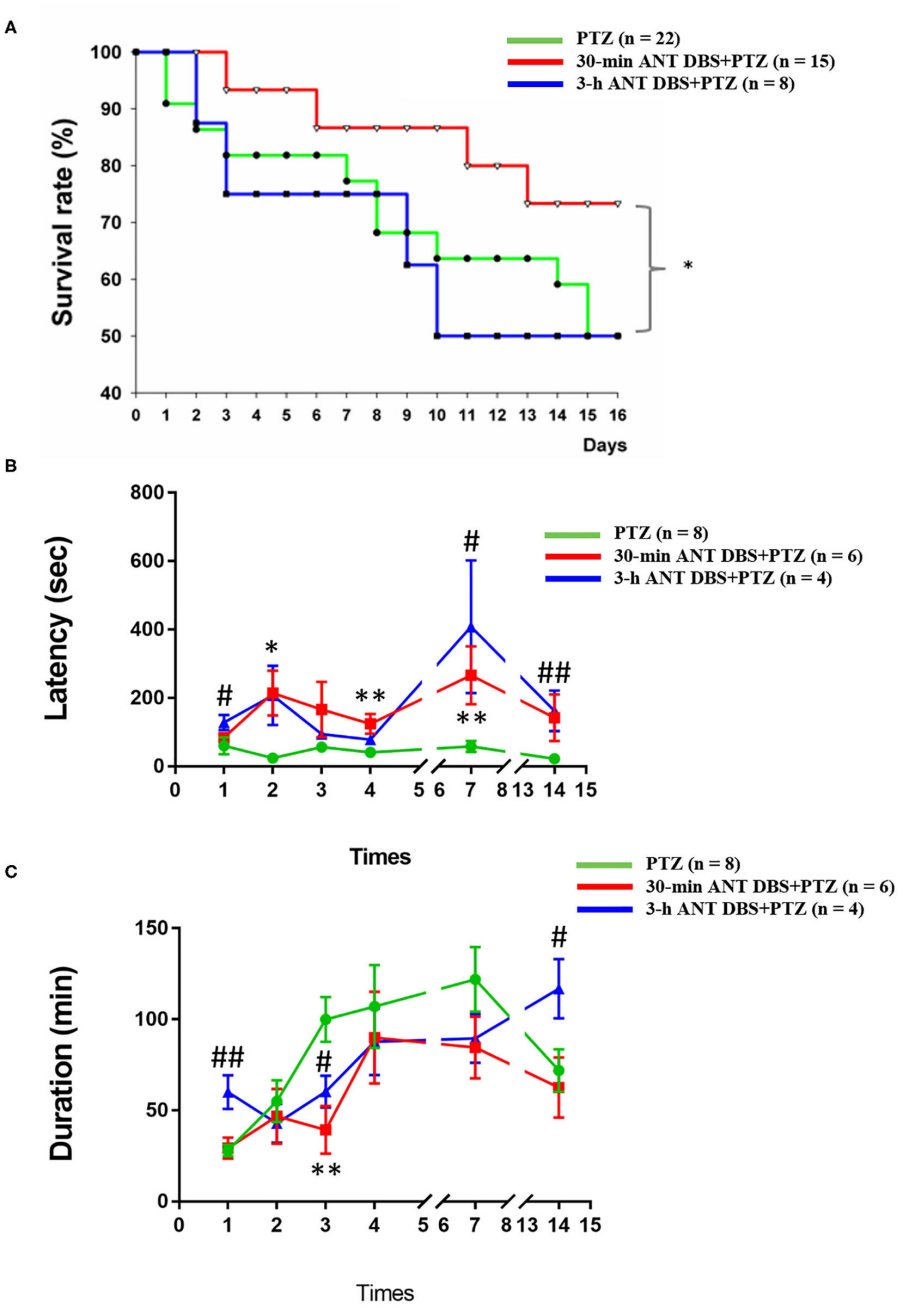
Effects of anterior nucleus of thalamus (ANT) deep brain stimulation (DBS) on the severity of pentylenetetrazol (PTZ)-induced seizure. **(A)** Effects of 30-min ANT DBS and 3-h ANT DBS on survival rate during the progression of PTZ kindling. *p < 0.05, 30-min ANT DBS + PTZ group vs. PTZ group, chi-square test. **(B)** ANT DBS prolonged the onset latency of ictal seizure induced by PTZ kindling. **(C)** ANT DBS reduced the duration of spontaneous seizure duration induced by PTZ kindling. *p < 0.05, **p < 0.01, 30-min ANT DBS + PTZ group vs. PTZ group, one-way ANOVA; ^#^*p* < 0.05, ^##^*p* < 0.01, 3-h ANT DBS + PTZ group vs. PTZ group, one-way ANOVA.

During establishing of fully kindled rats by 14 times of continuous PTZ injections, the severity of seizure increased and the survival rate decreased to 50%. In the 30-min ANT DBS + PTZ group, 30-min ANT DBS significantly rescued the survival rate from 50 to 73.3% after the 14th PTZ injection (*p* < 0.05, chi-square test), while 3-h DBS did not improve it (Figure 2A). The mortality rates for the PTZ group, the 30-min ANT DBS + PTZ group, and the 3-h ANT DBS + PTZ group were 50% (11 out of 22 rats died), 26.7% (four out of 15 rats died), and 50% (four out of eight rats died). However, the final sample sizes for each group were eight, six, and four rats for the PTZ group, the 30-min ANT DBS + PTZ group, and the 3-h ANT DBS + PTZ group because of the inaccurate electrode placement or loss of EEG headpiece.

Both 30-min and 3-h unilateral ANT DBS prolonged the latency to the onset of ictal epileptiform EEGs after the PTZ injections. The latencies to the onset of ictal epileptiform EEGs were 60.0 ± 24.7, 24.0 ± 7.5, 56.6 ± 15.8, 41.0 ± 14.1, 58.5 ± 16.3, and 22.5 ± 5.8 s (*n* = 8; Figure 2B) after the 1st, 2nd, 3rd, 4th, 7th, and 14th PTZ injections, respectively. Thirty minutes of DBS prolonged the onset latency to 84.0 ± 9.8, 214.3 ± 65.1 (*p* < 0.05, one-way ANOVA vs. 2nd PTZ injection), 166.3 ± 80.5, 124.0 ± 28.7 (*p* < 0.05, one-way ANOVA vs. 4th PTZ injection), 266.4 ± 84.7 (*p* < 0.05, one-way ANOVA vs. 7th PTZ injection), and 142.0 ± 68.1 s (*n* = 6; Figure 2B) after the 1st, 2nd, 3rd, 4th, 7th, and 14th PTZ injections, respectively. Three hours of DBS also prolonged the onset latency to 128.6 ± 22.3 (*p* < 0.05, one-way ANOVA vs. 1st PTZ injection), 207.4 ± 86.6, 94.0 ± 15.0, 78.0 ± 8.0, 408.0 ± 194.1 (*p* < 0.05, one-way ANOVA vs. 7th PTZ injection), and 162.0 ± 59.9 s (*p* < 0.05, one-way ANOVA vs. 14th PTZ injection) (*n* = 4; Figure 2B) after the 1st, 2nd, 3rd, 4th, 7th, and 14th PTZ injections, respectively.

Unilateral ANT DBS decreased the spontaneous seizure duration when compared with that obtained from PTZ kindling rats. The spontaneous seizure durations obtained after the 1st, 2nd, 3rd, 4th, 7th, and 14th PTZ injections were 28.0 ± 3.2, 55.0 ± 11.6, 100.0 ± 12.3, 107.1 ± 22.8, 121.9 ± 17.7, and 72.0 ± 11.7 min, respectively (*n* = 8; Figure 2C). Thirty minutes of unilateral ANT DBS reduced the spontaneous seizure duration to 29.3 ± 5.8, 46.8 ± 15.0, 39.3 ± 13.0 (*p* < 0.05, one-way ANOVA vs. 3rd PTZ injection), 90.0 ± 25.2, 84.6 ± 16.8, and 62.6 ± 16.5 min after the 1st, 2nd, 3rd, 4th, 7th, and 14th PTZ injections, respectively (*n* = 6; Figure 2C). Three hours of unilateral ANT DBS reduced the spontaneous seizure duration to 42.9 ± 10.7, 60.4 ± 8.8 (*p* < 0.05, one-way ANOVA vs. 3rd PTZ injection), 87.7 ± 18.3, and 89.5 ± 13.4 min after the 2nd, 3rd, 4th, and 7th PTZ injections, respectively (*n* = 4; Figure 2C). There was an unexpected increase of seizure duration after the 14th PTZ injection, which may be due to the agglomerated tissue debris at the tip of the DBS electrode after 14 times of 3-h ANT DBS and loss of DBS efficacy as we deal with later in the discussion.

Besides prolonging the onset latency to ictal seizure and shortening the duration of spontaneous seizure, both 30-min and 3-h unilateral ANT DBS only significantly reduced the ictal epileptiform amplitude from 947.6 ± 39.4 to 726.3 ± 70.5 μV (*p* < 0.05, one-way ANOVA) and 770.4 ± 56.5 μV (*p* < 0.05, one-way ANOVA), respectively, after the 2nd PTZ injection, while unilateral ANT DBS exhibited no effect on the reduction of ictal epileptiform amplitude during the progression of the kindling process (data not shown).

### Thirty Minutes of Unilateral ANT DBS Suppressed the PTZ-Induced Enhancement of the EEG Spectrum

**Figure 3:**
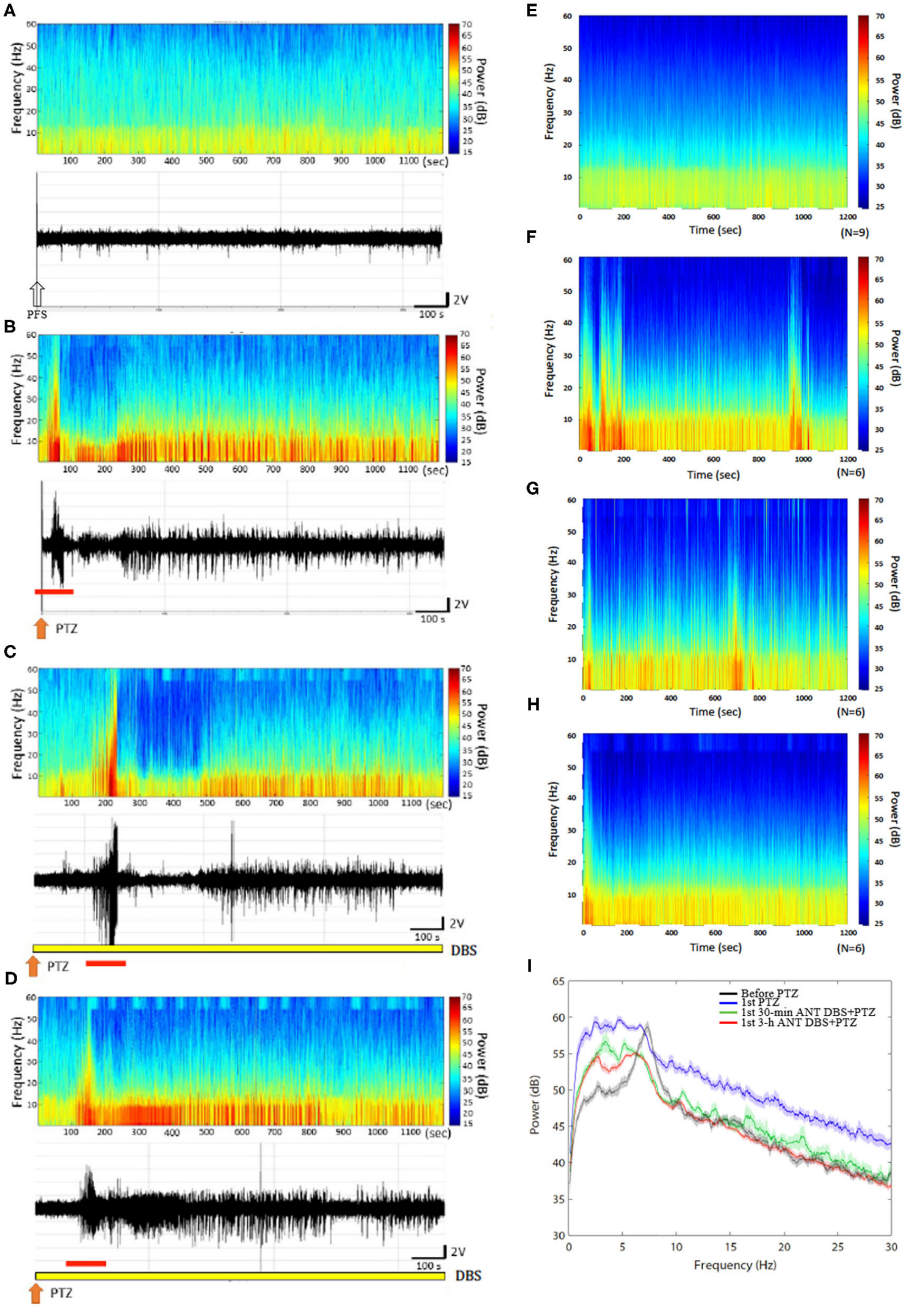
Anterior nucleus of thalamus (ANT) deep brain stimulation (DBS) suppressed the 1st pentylenetetrazol (PTZ) injection-induced enhancement of power spectrum. **(A)** The power spectrum and the raw electroencephalogram (EEG) obtained after the vehicle (pyrogen-free solution) injection in one rat. **(B)** The power spectrum and the raw EEGs acquired after the 1st PTZ injection in one rat. **(C)** The power spectrum and the raw EEGs obtained after the 1st 30-min ANT DBS + PTZ in one rat. **(D)** The power spectrum and the raw EEGs obtained after the 1st 3-h ANT DBS + PTZ in one rat. **(E)** The average of the power spectrum obtained from a group of rats after the vehicle injection. **(F)** The average of the power spectrum obtained from a group of rats after the 1st PTZ injection. **(G)** The average of the power spectrum obtained from a group of rats after the 1st 30-min ANT DBS + PTZ. **(H)** The average of the power spectrum obtained from a group of rats after the 1st 3-h ANT DBS + PTZ. **(I)** The summary of alterations in power spectrum. The white arrow represents the vehicle injection, the orange arrow depicts the injection of PTZ, the yellow bar represents the ANT DBS, and the red bar demonstrates the ictal epilepsy.

An analysis of the spectrograms of FFTs indicated that the intensities of all frequencies were enhanced during the 1st PTZ-induced ictal period and during the subsequent spontaneous seizure. Notably, the intensity of the low frequencies was significantly increased during the spontaneous seizure. The upper panels of Figures 3A,B, respectively, demonstrated the FFT spectrogram obtained from the control and after the first time of PTZ injection from one rat. The lower panels of Figures 3A,B depicted the EEGs obtained from the control and after the 1st PTZ injection. Figures 3E,F represented the average of the FFT spectrogram obtained before and after the first time of PTZ injection, respectively, in one group of rats. Thirty minutes of unilateral ANT DBS suppressed the augmentation of the low-frequency intensity during the spontaneous seizure induced by the 1st PTZ injection without altering the power intensity during the ictal seizure period (Figure 3C; the upper panel represents one example of rats; Figure 3G depicts the average in one group), while 3-h unilateral ANT DBS did not suppress the intensity of the low-frequency spectrum (Figure 3D; the upper panel represents one example of rats; Figure 3H depicts the average in one group). When analyzing the power of spectrum, the powers of all the spectra were increased after the 1st injection of PTZ, and both 30-min and 3-h ANT DBSs suppressed the enhancement (Figure 3I).

**Figure 4:**
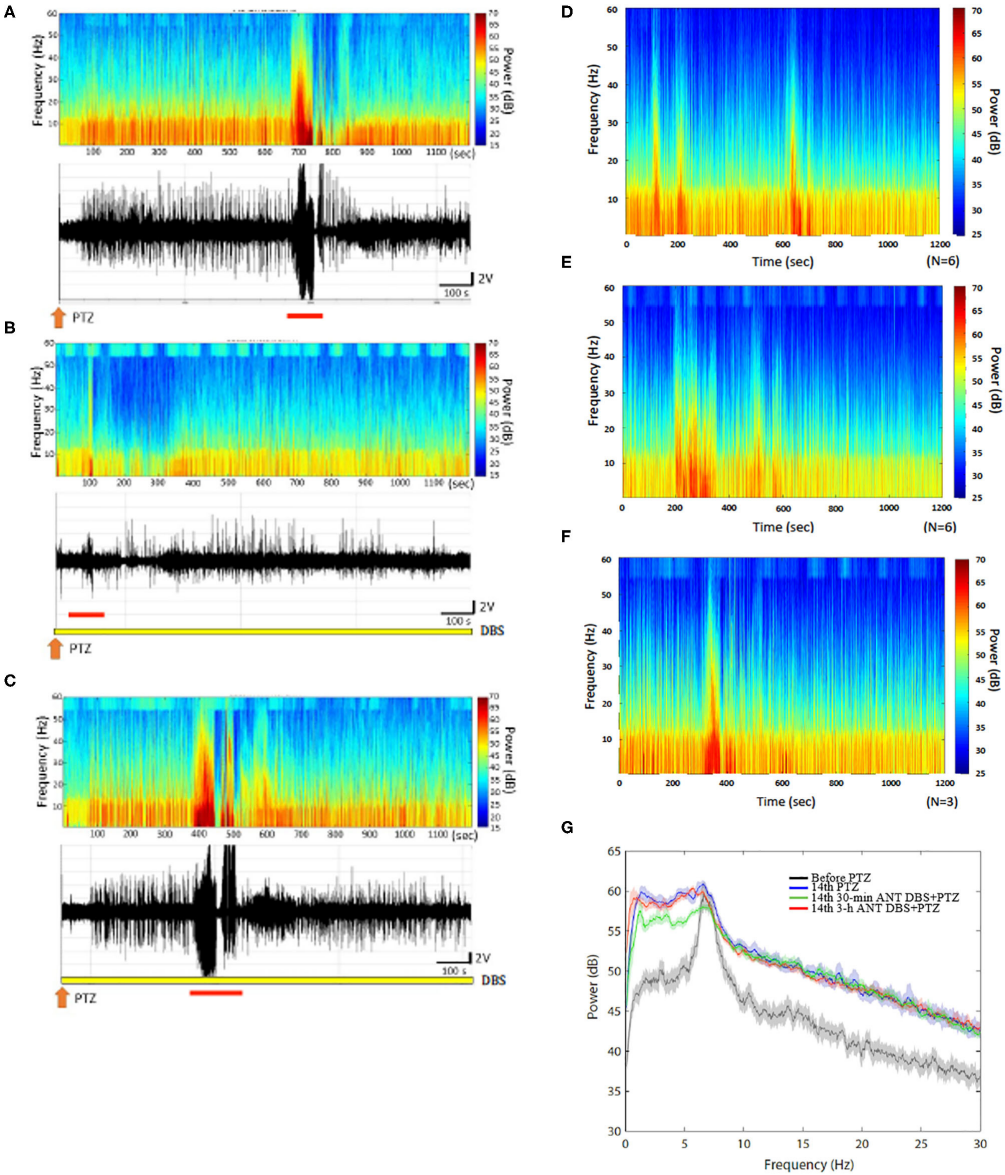
Anterior nucleus of thalamus (ANT) deep brain stimulation (DBS) suppressed the 14th pentylenetetrazol (PTZ) injection-induced enhancement of the power spectrum. **(A)** The power spectrum and the raw electroencephalogram (EEG) acquired after the 14th PTZ injection in one rat. **(B)** The power spectrum and the raw EEGs obtained after the 14th 30-min ANT DBS + PTZ in one rat. **(C)** The power spectrum and the raw EEGs obtained after the 14th 3-h ANT DBS + PTZ in one rat. **(D)** The average of the power spectrum obtained from a group of rats after the 14th PTZ injection. **(E)** The average of the power spectrum obtained from a group of rats after the 14th 30-min ANT DBS + PTZ. **(F)** The average of the power spectrum obtained from a group of rats after the 14th 3-h ANT DBS + PTZ. **(G)** The summary of alterations in the power spectrum. The orange arrow depicts the injection of PTZ, the yellow bar represents the ANT DBS, and the red bar demonstrates the ictal epilepsy.

Thirty minutes of unilateral ANT DBS reduced the power intensity of the low frequencies induced after the 14th PTZ injection. The upper panels of Figures 4A,B, respectively, represent the FFT of EEGs obtained after the 14th PTZ injection and the 14th time of 30-min ANT DBS + PTZ from one example of rats. Figures 4D,E depict the average of the FFT EEGs obtained from one group of rats after the 14th PTZ injection and the 14th time of 30-min ANT DBS + PTZ, respectively. Nevertheless, 3-h unilateral ANT DBS did not alter the power intensities of spontaneous seizure after the 14th PTZ injection (Figure 4C represents one example of rats; Figure 4F depicts the average in one group). When analyzing the power of spectrum, the powers of all the spectra were increased after the 14th injection of PTZ, and only 30-min ANT DBS suppressed the PTZ-kindling-induced enhancement of low-frequency powers, but not 3-h ANT DBS (Figure 4G).

### The Effects of ANT DBS on Sleep–Wake Activity

**Figure 5:**
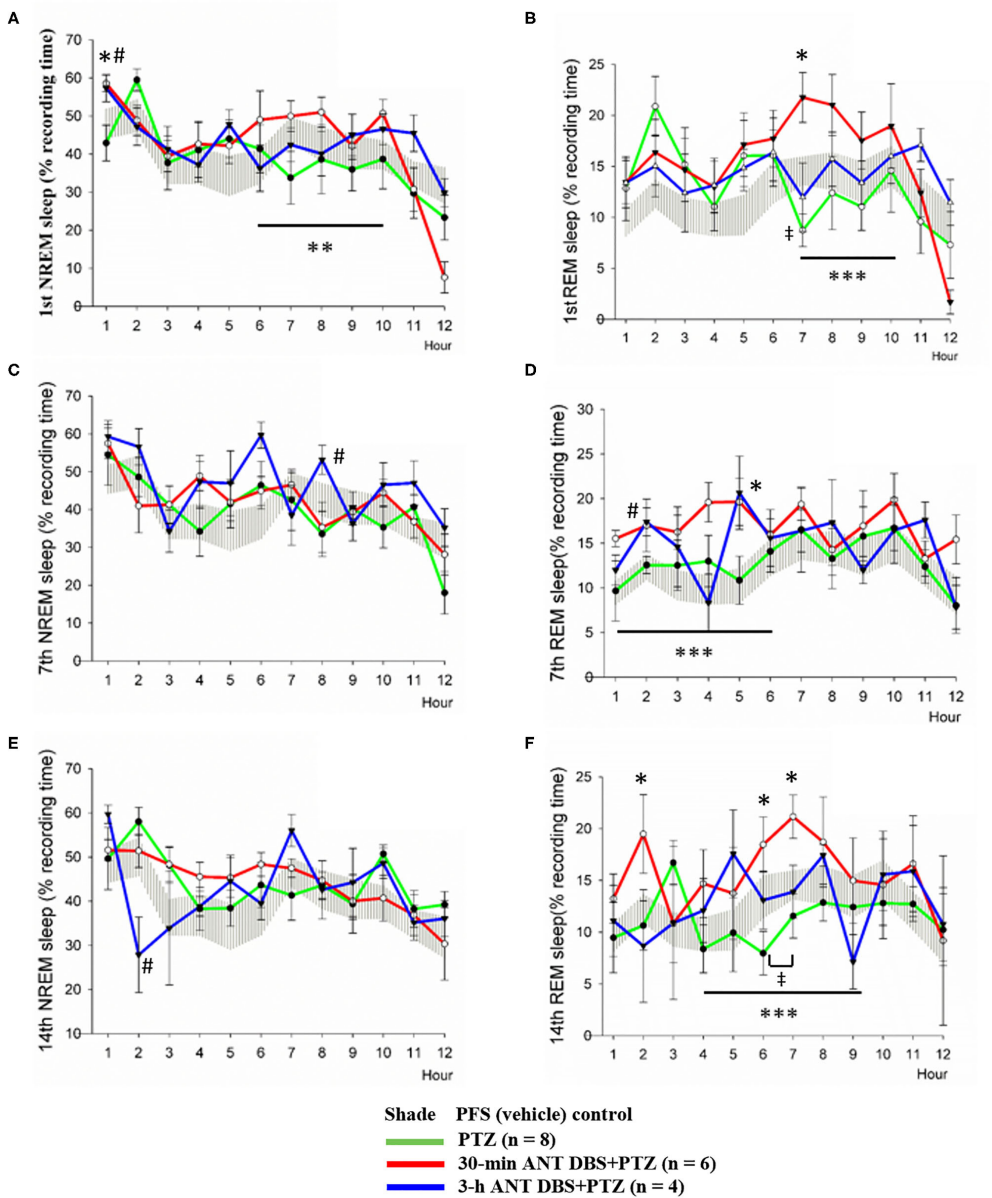
Effects of pentylenetetrazol (PTZ) and anterior nucleus of thalamus (ANT) deep brain stimulation (DBS) on non-rapid eye movement (NREM) sleep and rapid eye movement (REM) sleep. **(A)** NREM sleep obtained after the vehicle injection, after the 1st pentylenetetrazol (PTZ) injection, after the 1st 30-min ANT DBS + PTZ, and after the 1st 3-h ANT DBS + PTZ. **(B)** REM sleep obtained before the PTZ injection, after the 1st PTZ injection, after the 1st 30-min ANT DBS + PTZ, and after the 1st 3-h ANT DBS + PTZ. **(C)** NREM sleep obtained before the PTZ injection, after the 7th PTZ injection, after the 7th 30-min ANT DBS + PTZ, and after the 7th 3-h ANT DBS + PTZ. **(D)** REM sleep obtained before the PTZ injection, after the 7th PTZ injection, after the 7th 30-min ANT DBS + PTZ, and after the 7th 3-h ANT DBS + PTZ. **(E)** NREM sleep obtained before the PTZ injection, after the 14th PTZ injection, after the 14th 30-min ANT DBS + PTZ, and after the 14th 3-h ANT DBS + PTZ. **(F)** REM sleep obtained before the PTZ injection, after the 14th PTZ injection, after the 14th 30-min ANT DBS+PTZ, and after the 14th 3-h ANT DBS+PTZ. *p* < 0.05, PTZ group vs. vehicle control, one-way ANOVA; *p < 0.05, **p < 0.01, ***p < 0.001, 30-min ANT DBS + PTZ group vs. PTZ group, one-way ANOVA; ^#^*p* < 0.05, 3-h ANT DBS + PTZ group vs. PTZ group, one-way ANOVA.

Our result demonstrated that NREM sleep was not altered during the 12-h light period after the 1st, 7th, and 14th PTZ injections (Figures 5A,C,E); however, the amounts of REM sleep in hour 7 after the 1st PTZ injection and during hours 6 and 7 after the 14th PTZ injection were significantly reduced (Figures 5B,F). Thirty minutes of unilateral ANT DBS enhanced the percentage of time that the rats spent in NREM sleep after the 1st PTZ injection. The time spent in NREM sleep increased from 42.9 ± 12.5 to 58.6 ± 5.7% (*p* < 0.05, one-way ANOVA) during the 12-h light period when the rats received 30-min of unilateral ANT DBS (Figure 5A). The total amount of NREM sleep during hours 6–10 of the light period was also enhanced from 37.7 ± 3.1 to 48.6 ± 2.1% (*p* < 0.05, one-way ANOVA) after the 30-min unilateral ANT DBS (Figure 5A). Thirty minutes of unilateral ANT DBS also increased REM sleep from 8.7 ± 4.2 to 21.8 ± 6.5% (*p* < 0.05, one-way ANOVA) during hour 7 of the light period, and the total amount of REM sleep was also increased from 11.7 ± 1.5 to 19.8 ± 1.5% (*p* < 0.05, one-way ANOVA) during hours 7–10 of the light period (Figure 5B). Furthermore, 30-min unilateral ANT DBS progressively increased REM sleep after the 7th and 14th PTZ injections. The total amount of REM sleep was increased from 12.1 ± 1.0% obtained after the 7th PTZ injection to 17.3 ± 0.9% after the 7th 30-min ANT DBS + PTZ (*p* < 0.05, one-way ANOVA) during hours 1–6 of the light period (Figure 5D). The time spent in REM sleep was significantly increased during hours 2, 6, and 7 after the 14th 30-min ANT DBS + PTZ when compared with those obtained after the 14th PTZ injection. The total amount of REM sleep was increased from 10.5 ± 1.0% obtained after the 14th PTZ injection to 17.0 ± 1.1% after the 14th 30-min ANT DBS + PTZ (*p* < 0.05, one-way ANOVA) during hours 4–9 of the light period (Figure 5F).

Three-hour unilateral ANT DBS did not significantly altered NREM and REM sleep, except that NREM sleep during hour 8 and REM sleep during hour 2 were significantly increased after the 7th 3-h ANT DBS + PTZ (Figures 5C,D), and NREM sleep during hour 2 was decreased after the 14th 3-h ANT DBS + PTZ (Figure 5E).

### Unilateral ANT DBS Suppressed PTZ-Enhanced Delta Power of NREM Sleep

**Figure 6:**
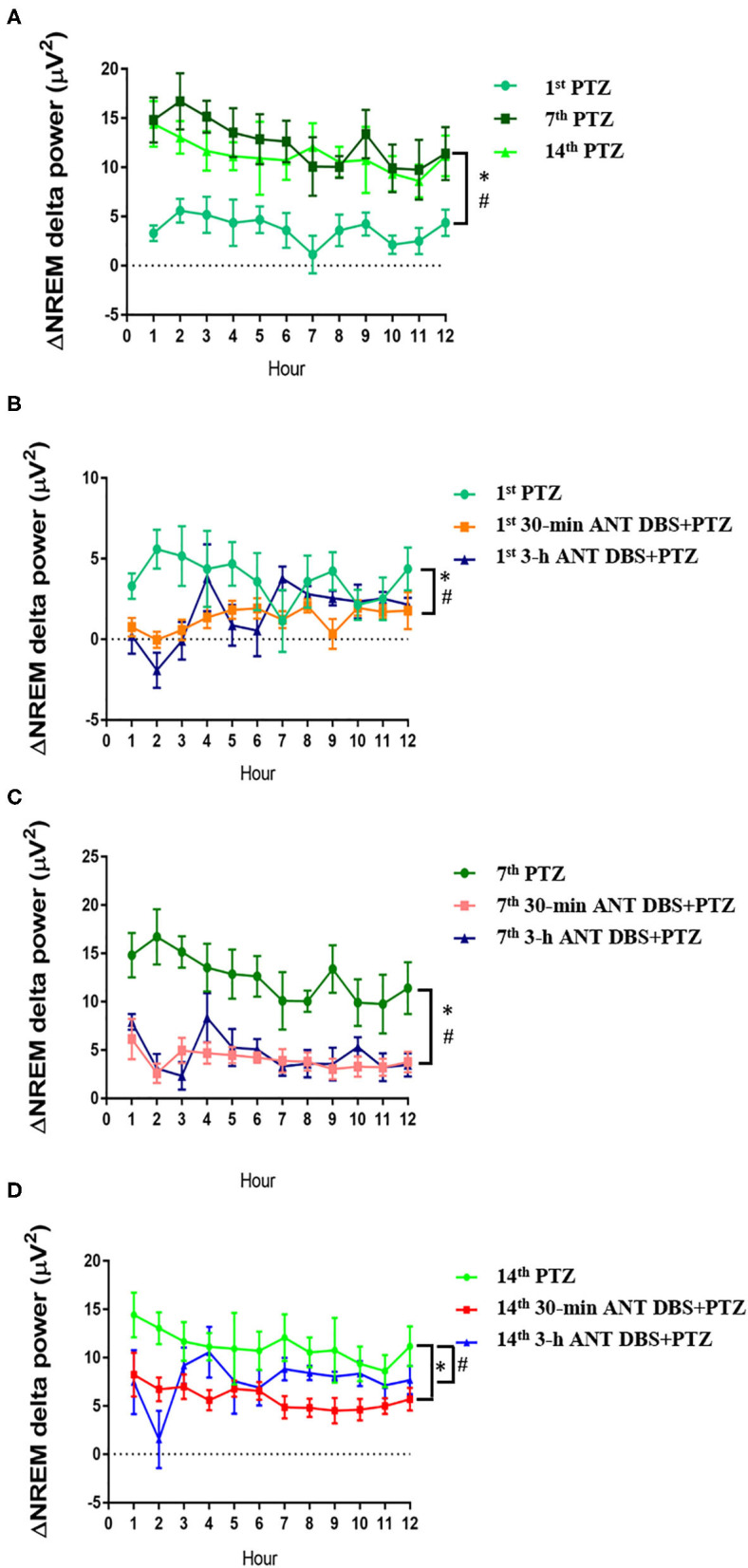
The alteration of delta power during non-rapid eye movement (NREM) sleep (ΔNREM delta power). **(A)** ΔNREM delta power obtained after the 1st, 7th, and 14th pentylenetetrazol (PTZ) injection comparing to that before the PTZ injection. *p < 0.05, 7th PTZ vs. 1st PTZ; ^#^*p* < 0.05, 14th PTZ vs. 1st PTZ, one-way ANOVA. **(B)** ΔNREM delta power acquired after the 1st PTZ injection, the 1st 30-min anterior nucleus of thalamus (ANT) deep brain stimulation (DBS) + PTZ, and the 1st 3-h ANT DBS + PTZ. *p < 0.05, 1st 30-min ANT DBS + PTZ group vs. 1st PTZ; ^#^*p* < 0.05, 1st 3-h ANT DBS + PTZ group vs. 1st PTZ, one-way ANOVA. **(C)** ΔNREM delta power acquired after the 7th PTZ injection, the 7th 30-min ANT DBS + PTZ, and the 7th 3-h ANT DBS + PTZ. *p < 0.05, 7th 30-min ANT DBS + PTZ group vs. 7th PTZ; ^#^*p* < 0.05, 7th 3-h ANT DBS + PTZ group vs. 7th PTZ, one-way ANOVA. **(D)** ΔNREM delta power acquired after the 14th PTZ injection, the 14th 30-min ANT DBS + PTZ, and the 14th 3-h ANT DBS + PTZ. *p < 0.05, 14th 30-min ANT DBS + PTZ group vs. 14th PTZ; ^#^*p* < 0.05, 14th 3-h ANT DBS + PTZ group vs. 14th PTZ, one-way ANOVA.

Consecutive injections of PTZ progressively increased the delta power during NREM sleep when compared with that acquired after an i.p. vehicle injection. The differences of delta power during NREM sleep (ΔNREM delta power) were, respectively, increased by 3.62 ± 0.43, 12.42 ± 0.70, and 11.20 ± 0.61 μV^2^ after the 1st, 7th, and 14th PTZ injections during the 12-h light period (Figure 6A). The ΔNREM delta powers obtained after the 7th and 14th PTZ injections were significantly higher than that acquired after the 1st PTZ injection (*p* < 0.05, one-way ANOVA; Figure 6A). Both 30-min and 3-h unilateral ANT DBS decreased the enhancement of ΔNREM delta powers induced by PTZ kindling. Thirty minutes of unilateral ANT DBS, respectively, decreased the ΔNREM delta power to 1.27 ± 0.18 μV^2^ (*p* < 0.05, one-way ANOVA vs. 1st PTZ injection), 4.00 ± 0.32 μV^2^ (*p* < 0.05, one-way ANOVA vs. 7th PTZ injection), and 5.86 ± 0.35 μV^2^ (*p* < 0.05, one-way ANOVA vs. 14th PTZ injection; Figures 6B–D) after the 1st, 7th, and 14th PTZ injections. Three hours of unilateral ANT DBS, respectively, decreased the ΔNREM delta power to 1.65 ± 0.44 μV^2^ (*p* < 0.05, one-way ANOVA vs. 1st PTZ injection), 4.53 ± 0.55 μV^2^ (*p* < 0.05, one-way ANOVA vs. 7th-PTZ injection), and 7.64 ± 0.74 μV^2^ (*p* < 0.05, one-way ANOVA vs. 14th PTZ injection; Figures 6B–D) after the 1st, 7th, and 14th PTZ injection.

## Discussion

In the current study, we established an epilepsy model in rats which spent less time to develop full kindling effects and a spontaneous recurrence of seizure with a sub-threshold dose of PTZ (40 mg/kg, i.p.). Our preliminary study demonstrated that the PTZ kindling protocol used in mice (25) cannot be reproduced in rats. It has been reported that the PTZ kindling protocol applied in rats needs more than 20 days with a lower dose of PTZ (26) or less time with a higher dose of PTZ (27). Our preliminary study elucidated that giving doses higher than 50 mg/kg PTZ caused a higher mortality rate (data not shown), and we chose an optimal dose of 40 mg/kg PTZ, which is the highest sub-threshold dose, by i.p. injection once a day for consecutive 14 days to induce full kindling of epilepsy. After the 7th PTZ injection, the spontaneous recurrence of spike-and-waves could be observed during NREM sleep 6 h after the injection, but the susceptibility of PTZ was different from rat to rat because of individual differences. Most of the rats can develop a spontaneous recurrence of epileptic spike-and-waves during wakefulness and NREM sleep after the 14th PTZ injection. Therefore, we employed 14 times of PTZ injections to establish a full kindling ictal period and spontaneous seizure recurrence in the current study.

DBS has been applied to treat Parkinson's disease (28) and chronic pain (29). There are several targets of DBS which exhibit efficacy in suppressing seizure, including the anterior thalamus (30), the centromedian thalamic nucleus (31), the caudate nucleus (32), the posterior hypothalamus (33), the hippocampus (34), and the subthalamic nucleus (35). The ANT plays a crucial role in seizure propagation since it, respectively, receives afferents from the mammillary bodies and subiculum, through the mammillothalamic tract and fornix, and projects to the parahippocampal gyrus and entorhinal cortex *via* the prefrontal cortex and cingulate gyrus (36, 37). Although several studies indicate a bilateral high-frequency stimulation of ANT or a lesion of ANT exhibiting suppression of occurrence of seizure and status epilepticus (19, 38), our previous result demonstrated that unilateral left ANT DBS with high-frequency (200 Hz) and low-intensity (50 μA) currents can successfully suppress pilocarpine-induced ictal and status epilepticus (16). Our previous study also suggests that unilateral ANT DBS should be delivered at least 30 min prior to pilocarpine administration to reduce ictal seizures and status epilepticus (16). In the current study, we tried to further elucidate the efficacy and the underlying mechanisms for unilateral ANT DBS on PTZ kindling-induced ictal seizure and spontaneous recurrence of seizure. Our result indicated that the latencies of ictal seizure onset were significantly prolonged by both 30-min and 3-h unilateral ANT DBS. The 30-min unilateral ANT DBS significantly prolonged the onset latency after the 2nd, 4th, and 7th PTZ injections, while the 3-h DBS prolonged the latency after the 1st, 7th, and 14th PTZ injections. Unilateral ANT DBS only reduced the duration of spontaneous seizure recurrence between the 3rd and 7th PTZ injections, especially that there was a significant reduction of spontaneous seizure duration after the 3rd PTZ injection. Thirty minutes of unilateral ANT DBS significantly increased the survival rate after the 14th PTZ injection when compared with the PTZ control group, while 3-h ANT DBS did not rescue the fatal rate from the PTZ full kindling of epilepsy. These results suggested that 30-min unilateral ANT DBS has a better efficacy in the suppression of PTZ-induced spontaneous seizure than 3-h ANT DBS. We have previously shown that unilateral ANT DBS started 1 h before the pilocarpine injection exhibited better suppressive effects in pilocarpine-induced ictal seizures than that of ANT DBS applied 30 min before the injection (16). However, 3-h ANT DBS did not show a better efficacy in spontaneous seizure suppression than that of 30-min ANT DBS in this study. When we sacrificed the rats and examined the tip of the DBS electrode, there was tissue debris agglomerated at the tip of the DBS electrode after 14 times of 3-h ANT DBS, while the agglomerated tissue debris was not found after the 14th 30-min ANT DBS + PTZ. This agglomerated tissue may block the currents delivered from the electrode to the tissue and weaken the efficacy of ANT DBS. This finding is also observed in another study which demonstrates that the tissue surrounding the electrode appears as a 1-mm-thick capsule around the electrode after a long-term DBS of the subthalamic nucleus (39). This agglomerated tissue debris at the tip of the DBS electrode after a longer time of stimulation may be due to the stimulation pattern and parameter *per se* and/or the immune response to the electrode (39). There may have been residues of free charges at the tip of the electrode after the stimulation to cause the agglomeration of brain tissue surround the tip. In a future study, we will artificially neutralize the charges on the tip of the electrode every time after the DBS stimulation to confirm this. We will also revise our stimulating protocol with injecting a neutralizing current after each stimulus to reduce tissue agglomeration. Determining the number of activated microglia and related cytokine expression would answer whether the lessened efficacy of 3-h DBS is caused by immune response.

Recent evidence demonstrates epilepsy results from the synchronization of neuronal firing within the neuronal circuits (40). The synchronous EEG oscillations during SWS play an important role in promoting the propagation of epileptiform EEGs. Asynchronous EEG oscillations during wakefulness and REM sleep, on the other hand, may suppress epileptogenesis (8). An analysis of the spectrograms of FFTs in the current study indicated that the intensities of all frequencies were enhanced during the PTZ-induced ictal seizure period, and the low frequencies (<10 Hz) were also increased during the subsequent spontaneous seizure. The increases of the low-frequency EEGs after the PTZ kindling include the delta (0.5–4 Hz) and theta (4–7 Hz) frequency bands. Unilateral ANT DBS significantly blocked the augmentation of the low-frequency band intensity of EEGs, the delta power and the theta power, during the spontaneous recurrence of seizure induced by PTZ kindling. Since ANT DBS *per se* exerts no effect on the power spectrum of unmanipulated EEGs during baseline as we have described previously (16), the blockade of the augmented low-frequency band intensity of EEGs by unilateral ANT DBS is a specific effect to suppress PTZ-induced seizure. The effect of ANT DBS in reducing EEG synchronization in delta and theta frequency bands to suppress seizure is similar to some effects of AEDs, such as the antiepileptic effect of valproate on generalized epilepsy (41). Sleep seems to become a key factor in the management of epilepsy since there is a vicious cycle between epilepsy and sleep—seizure may cause sleep disturbance, which worsens the recurrence of seizure. Although many studies conclude that AEDs, vagus nerve stimulation, and a ketogenic diet affects sleep during seizure treatment (42–44), very few studies addressed this issue for ANT DBS. One study indicates that DBS of the anterior thalamic nucleus disrupts sleep in epilepsy patients (45). Our results demonstrated that PTZ kindling-induced seizure did not alter NREM sleep; however, the amounts of REM sleep in hour 7 after the 1st PTZ injection and during hours 6 and 7 after the 14th PTZ injection were significantly reduced. Unilateral ANT DBS did not significantly alter NREM sleep but increased the amounts of REM sleep. The ANT DBS only slightly increased NREM sleep after the first time of DBS stimulation, while the total amounts of REM sleep after the 1st, 7th, and 14th injections of 30-min ANT DBS were significantly enhanced, but REM sleep was not altered by 3-h ANT DBS. Since ANT DBS *per se* exerts no effect on sleep–wake activity as we have reported previously (16), the increase of REM sleep to desynchronize EEG oscillations may be one of the mechanisms for the 30-min ANT DBS to suppress the spontaneous recurrence of seizure. Nevertheless, the direct evidence to prove that the underlying mechanism of ANT DBS on seizure suppression is mediated by enhancement of REM sleep needs more experiments, such as manipulation of REM sleep during ANT DBS, for further confirmation.

Delta powers during NREM sleep may represent as a biomarker for recurrent seizures. Patients with recurrent seizures showed significantly increased delta activities in magnetoencephalography and intracranial electroencephalography (46). A progressive enhancement of delta powers during NREM sleep was found after 14 consecutive times of PTZ injections in this study, which further confirms that increases of delta synchronization during NREM sleep reduce the seizure threshold to initiate a spontaneous recurrence of epilepsy. Electrical stimulation of the central lateral nucleus of the intralaminar thalamus and the pontine nucleus oralis causes suppression of delta waves in the cortex and restores consciousness in epileptic rats (47). Our current study also demonstrated that both 30-min and 3-h ANT DBSs significantly decrease the PTZ kindling-induced enhancement of delta power during NREM sleep. As we have mentioned previously, synchronous EEG oscillations during SWS play an important role in promoting the propagation of epileptiform EEGs (8). The reduction of delta powers during NREM sleep may reduce the probability of a spontaneous recurrence of seizure.

In conclusion, our results elucidated that unilateral ANT DBS enhanced the seizure threshold by increasing the amounts of REM sleep and decreasing the progressive enhancement of delta power during NREM sleep to suppress spontaneous seizure recurrences in PTZ kindling-induced epileptic rats.

## Data Availability Statement

All datasets generated for this study are included in the article/supplementary material.

## Ethics Statement

The animal study was reviewed and approved by National Taiwan University Institutional Animal Care and Use Committee.

## Author Contributions

H-TT, P-LY, and F-CC designed the experiment. H-TT and Y-TH collected and analyzed the data. P-LY and F-CC wrote the manuscript. H-TT, Y-TH, and F-CC prepared Figures 1–6. All the authors reviewed the manuscript.

## Conflict of Interest

The authors declare that the research was conducted in the absence of any commercial or financial relationships that could be construed as a potential conflict of interest.
